# Outcomes of viral coinfections in infants hospitalized for acute bronchiolitis

**DOI:** 10.1186/s12985-023-02197-7

**Published:** 2023-10-16

**Authors:** Lorena Bermúdez-Barrezueta, Pablo López-Casillas, Silvia Rojo-Rello, Laura Sáez-García, José Manuel Marugán-Miguelsanz, María de la Asunción Pino-Vázquez

**Affiliations:** 1 División of Pediatric and Neonatal Intensive Care, Department of Pediatrics, Hospital Clínico Universitario de Valladolid, Av. Ramón y Cajal, 3, 47003 Valladolid, Spain; 2 Department of Pediatrics, Faculty of Medicine, Valladolid University, Valladolid, Spain; 3 Microbiology and Immunology Department, Hospital Clínico Universitario de Valladolid, Valladolid, Spain; 4 Division of Pediatric Intensive Care, Reina Sofía Hospital, Córdoba, Spain; 5 Division of Gastroenterology and Pediatric Nutrition, Head of Department of Pediatrics, Hospital Clínico Universitario de Valladolid, Valladolid, Spain

**Keywords:** Acute bronchiolitis, Viral coinfection, Severity, Length of stay, Pediatric intensive care unit.

## Abstract

**Background and Objective:**

The clinical relevance of the detection of multiple respiratory viruses in acute bronchiolitis (AB) has not been established. Our goal was to evaluate the effect of viral coinfections on the progression and severity of AB.

**Methods:**

A retrospective observational study was conducted in a tertiary hospital in Spain from September 2012 to March 2020. Infants admitted for AB with at least one respiratory virus identified by molecular diagnostic techniques were included. A comparison was made between single-virus infections and viral coinfections. The evolution and severity of AB were determined based on the days of hospitalization and admission to the pediatric intensive care unit (PICU).

**Results:**

Four hundred forty-five patients were included (58.4% male). The median weight was 5.2 kg (IQR 4.2–6.5), and the median age was 2.5 months (IQR 1.4–4.6). A total of 105 patients (23.6%) were admitted to the PICU. Respiratory syncytial virus (RSV) was the most frequent etiological agent (77.1%). A single virus was detected in 270 patients (60.7%), and viral coinfections were detected in 175 (39.3%), of which 126 (28.3%) had two viruses and 49 (11%) had three or more viruses. Hospital length of stay (LOS) increased in proportion to the number of viruses detected, with a median of 6 days (IQR 4–8) for single infections, 7 days (IQR 4–9) for coinfections with two viruses and 8 days (IQR 5–11) for coinfections with ≥ 3 viruses (p = 0.003). The adjusted Cox regression model showed that the detection of ≥ 3 viruses was an independent risk factor for a longer hospital LOS (HR 0.568, 95% CI 0.410–0.785). No significant association was observed between viral coinfections and the need for PICU admission (OR 1.151; 95% CI 0.737–1.797).

**Conclusions:**

Viral coinfections modified the natural history of AB, prolonging the hospital LOS in proportion to the number of viruses detected without increasing the need for admission to the PICU.

## Significance

**What is Known**

The main etiological agent of acute bronchiolitis (AB) is respiratory syncytial virus (RSV); however, other viruses are frequently detected. All viruses may be the sole etiological cause or may occur in association, and a high prevalence of viral coinfection has been described.

To date, there are conflicting results on the role of viral coinfections in the severity of bronchiolitis.

**What is New**

Viral coinfections influence the progression of AB. The simultaneous detection of 3 or more respiratory viruses is a risk factor for longer hospital stay.

The presence of viral coinfections does not condition a greater need for admission to the PICU.

## Introduction

Acute bronchiolitis (AB) is the most common lower respiratory infection in infants and a major cause of hospitalization, especially in children under 6 months of age, with a greater prevalence in the autumn and winter months [1].

The main etiologic agent of AB is respiratory syncytial virus (RSV); however, 10 to 20% of cases may be caused by other viruses. Among these, viruses of the family *Picornaviridae*, influenza viruses and adenoviruses, among others, have classically been involved [2]. However, the emergence in recent years of new molecular techniques has improved the detection of viral agents over conventional techniques such as shell-vial viral culture or direct immunofluorescence and has provided insights into the detection of novel viral pathogens such as human metapneumovirus, bocavirus and coronavirus [3–5].

Respiratory viral coinfections, defined as the detection of more than one viral pathogen in the same specimen, are detected in 10–55% of children with acute respiratory tract infection [5–11]. RSV and rhinovirus remain the most frequently associated viruses in viral coinfections [12, 13]. To date, there are conflicting results in the different studies that have tried to determine if the presence of viral coinfections causes greater severity in patients with AB. The objective of the present study was to evaluate the effect of viral coinfections on the progression and severity of acute bronchiolitis (AB) by hospitalization days and admission to the pediatric intensive care unit (PICU).

## Methods

A retrospective observational study was conducted in a cohort of patients under 2 years of age admitted for AB at the Hospital Clínico Universitario de Valladolid between September 1, 2012, and March 15, 2020, before the onset of the COVID-19 pandemic. We included children diagnosed with AB for which at least one virus was identified by molecular diagnostic techniques in nasopharyngeal lavage or pharyngeal smear samples collected during the first 24 h of admission. Patients who did not undergo an etiological study with molecular tests were excluded.

### Detection of respiratory viruses

Two molecular techniques were used for etiological diagnosis: the Luminex® NxTAG Respiratory Pathogen PCR Panel (NxTAG RPP) and FilmArray® Respiratory Panel.

The Luminex® NxTAG RPP is a qualitative molecular test for the identification of nucleic acids from 17 respiratory viruses and 3 bacteria: adenovirus, coronavirus (229E, HKU1, OC43 and NL63), bocavirus, human metapneumovirus, human rhinovirus/enterovirus, influenza virus (A, A/H1-2009, A/H3, B), parainfluenza (1, 2, 3 and 4), RSV, *Legionella pneumophila, Chlamydophila pneumoniae* and *Mycoplasma pneumoniae*. The sensitivity and specificity of this test is greater than 95% [14, 15]. The Luminex® NxTAG RPP was used according to the instructions in the product leaflet with an approximate duration of 4–5 h.

The FilmArray® Respiratory Panel analyses 17 viruses and 3 bacteria that cause respiratory tract infections, with a sensitivity and specificity of 95% and 99%, respectively. This is a multiplex PCR system with a total runtime of less than one hour. The panel includes the following targets: adenovirus, coronavirus (229E, HKU1, OC43, NL63, MERS), human metapneumovirus, human rhinovirus/enterovirus, influenza virus (A, A/H1 2009, A/H3, B), bocavirus, parainfluenza (1, 2, 3 and 4), RSV, *Bordetella pertussis*, *Chlamydophila pneumoniae* and *Mycoplasma pneumoniae* [16]. This technique was carried out following the manufacturer’s instructions and was used in selected cases in which the urgency of the etiological diagnosis allowed decisions to be made concerning therapeutic management.

### Data collection

Data collection was carried out by reviewing medical records, and the identification of the diagnosis was obtained from the Clinical Documentation and Archives Unit, which provided a registry of children with AB at discharge, according to the International Classification of Diseases, 9th Revision, Clinical Modification (ICD-9-CM) during the years 2012–2015, with the following codes: ICD-9 466.1 Acute Bronchiolitis, ICD-9 466.11 RSV Bronchiolitis, and ICD-9 466.19 Acute Bronchiolitis due to other infectious organisms. Since 2016, the International Classification of Diseases, 10th Revision (ICD-10) has been used, with the following codes: ICD-10 J21.0 Acute bronchiolitis due to RSV, ICD-10 J21.1 Acute bronchiolitis due to human metapneumovirus, ICD-10 J21.8 Acute bronchiolitis due to other specified organisms and ICD-10 J21.9 Acute bronchiolitis not specified.

Based on the McConnochie criteria, the first episode of respiratory distress that occurred with wheezing and/or crackles or rales, preceded by a catarrhal stage, that affected children under 2 years of age was considered AB [17]. Cases that did not meet the above criteria were excluded.

Viral coinfection was defined as the simultaneous identification of two or more viruses in a single respiratory sample. In addition, the number of viral associations detected in each patient was recorded.

A comparison was made between single-virus infections and viral coinfections. The progression and severity of AB were determined based on the days of hospitalization and admission to the PICU. In addition, other variables were collected, such as sex, gestational age, birth weight, type of delivery, age and weight at admission, family history of atopy (asthma in parents or siblings), personal history of atopy (allergic rhinitis, atopic dermatitis, food or pharmacological allergy), siblings, underlying diseases, breastfeeding, maximum C-reactive protein level, clinical manifestations, sepsis with microbiological confirmation, antibiotherapy and respiratory support such as low-flow oxygen therapy, high-flow nasal cannula (HFNC) oxygen therapy, noninvasive ventilation (NIV) and invasive mechanical ventilation (IMV). Data were collected by the research team.

This study was performed in line with the principles of the Declaration of Helsinki. Approval was granted by the Ethics Committee of the Hospital Clínico Universitario de Valladolid, Spain (PI 20-1902).

### Statistical analysis

Data analysis was performed using IBM SPSS 24.0 (SPSS Inc., Chicago, Illinois, United States). Categorical variables are expressed as absolute values and percentages, and quantitative variables are expressed as the means and standard deviations (SDs) if they were normally distributed (Kolmogorov–Smirnov and/or Shapiro–Wilk test) or as the medians and interquartile ranges (IQRs) if they were not normally distributed. For the analysis of the categorical variables, the chi-square or Fisher’s exact test was used; continuous variables were analyzed with the Kruskal‒Wallis, Mann‒Whitney U or Student *t* test, according to the normality of the data.

The relationship between viral coinfections and admission to the PICU was evaluated by univariate and multivariate logistic regression analyses with a stepwise backward elimination procedure. The results are presented as odds ratios (ORs) and 95% confidence intervals (95% CIs).

To assess the independent effect of the number of viral associations on the hospital length of stay (LOS), univariate and multivariate analyses were performed with Cox proportional hazards regression models with the stepwise backward elimination method. The results are presented as hazard ratios (HRs) and 95% CIs. The primary event was defined as “hospital discharge”, and the speed with which the event occurred during the period studied was assessed (HR < 1 = lower speed and longer length of hospital stay).

Variables with p < 0.2 in the univariate analysis and/or those considered clinically important for the control of confounders were included in the multivariate models. A p value < 0.05 was considered statistically significant.

## Results

During the study period, 485 patients diagnosed with AB were admitted, and 467 molecular diagnostic tests were performed for the detection of respiratory viruses, with a positive result in 95.3% of cases. We included 445 patients from whom at least one respiratory virus was isolated; 58.4% were male. The median weight at admission was 5.2 kg (IQR 4.2–6.5), and the median age was 2.5 months of age (IQR 1.4–4.6). One hundred five patients.

(23.5%) were admitted to the PICU. The median hospital LOS was 6 days (IQR 4–9). The sociodemographic and clinical characteristics of the patients are shown in Table 1.

**Table 1:** Sociodemographic and clinical characteristics at admission of infants included in the study

	n = 445
Sex (% male)	260 (58.4)
Gestational age (weeks)	39 [37–40]
Birth weight (grams)	3100 [2745–3440]
Weight at admission (Kilograms)	5.2 [4.2–6.5]
Age at admission (months)	2.5 [1.4–4.6]
Prematurity (%)	67 (15.1)
Type of delivery (% caesarean section)	143 (32.1)
Patients with at least 1 comorbidity (%)	35 (7.9)
Underlying disease (%)	
Chronic lung disease Airway anomaly Congenital heart disease Central nervous system disorder Neuromuscular disease Down syndrome Others	4 (0.9) 2 (0.5) 24 (5.4) 6 (1.3) 2 (0.5) 3 (0.7) 2 (0.5)
Family history of atopy (%)	84 (18.9)
Personal history of atopy (%)	33 (7.4)
Siblings (%)	289 (64.9)
Feeding modality (%)	
Breastfeeding Formula feeding	251 (56.4) 180 (40.4)
Fever (%)	222 (49.9)
Food refusal (%)	257 (57.8)
Apneas (%)	25 (5.6)

Categorical variables expressed as absolute frequency and percentage, and quantitative variables as median and interquartile range [IQR].

**Fig. 1:**
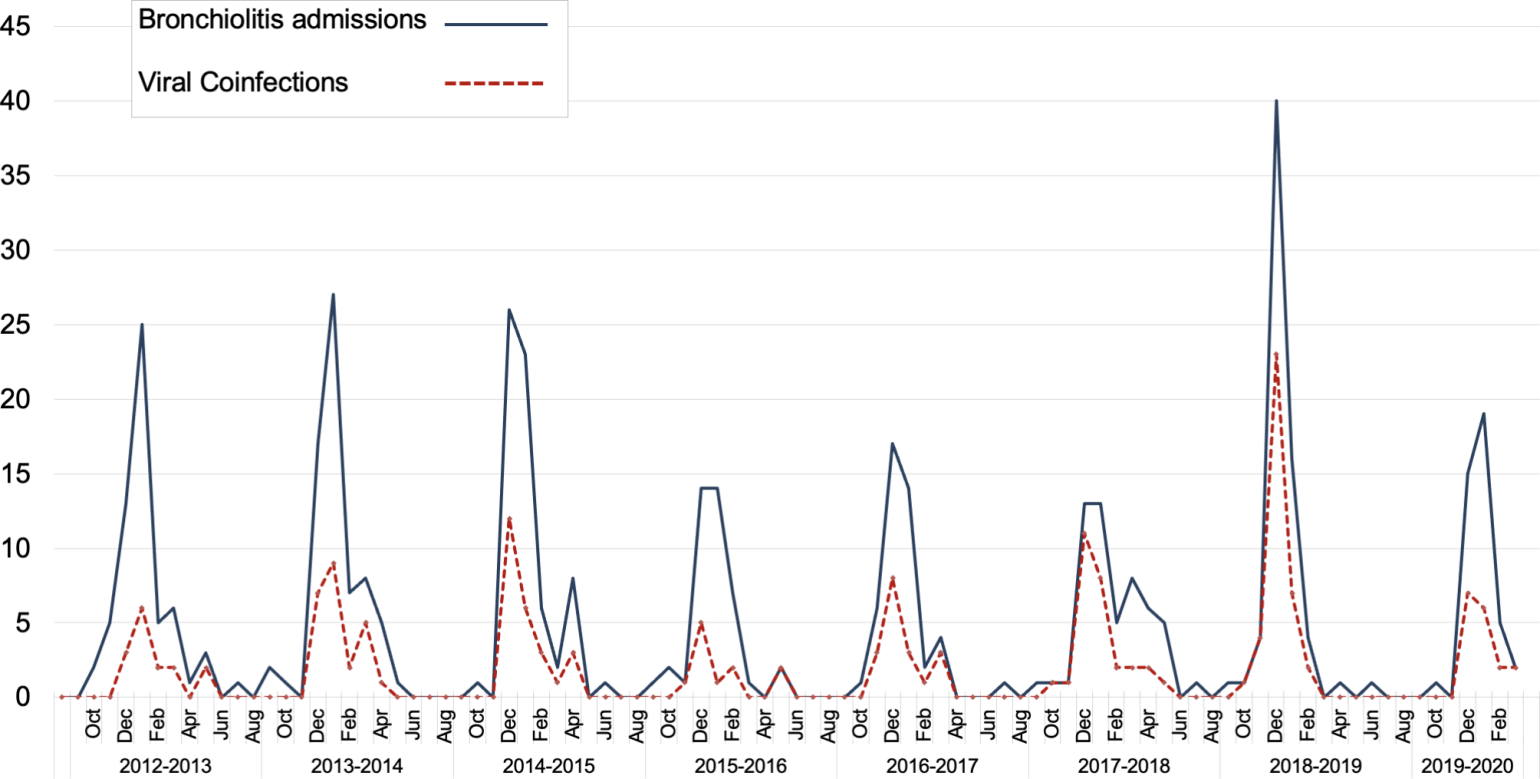
Monthly distribution of bronchiolitis admissions (blue line) and viral coinfection admissions (dashed red line) during each epidemic season

Concerning viral etiology, we observed that a single virus was detected in 270 patients (60.7%) and viral coinfections in 175 (39.3%), of which two viruses were identified in 126 (28.3%) and three or more viruses were identified in 49 (11%). Figure 1 shows the distribution of monthly admissions for AB in which at least one respiratory virus was isolated and the frequency of viral coinfections.

**Fig. 2:**
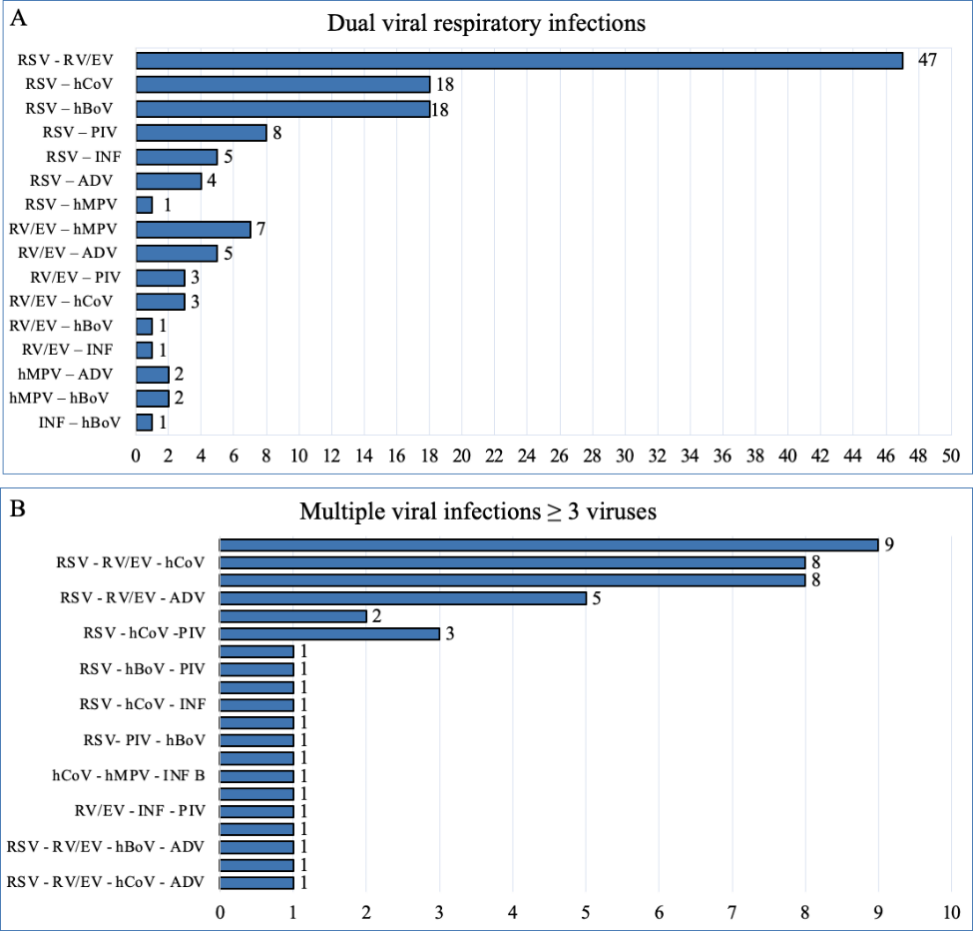
Viral coinfections detected in hospitalized bronchiolitis patients (absolute number). **A** dual viral infection; **B** multiple viral infections *RSV* respiratory syncytial virus, *RV/EV* rhinovirus/enterovirus, *hCoV* coronavirus (229E, HKU1, OC43 and NL63), *hBoV* bocavirus, *PIV* parainfluenza (1, 2, 3 and 4), *INF* influenza (A, A/H1, A/H1-2009, and A/H3) *INF B* influenza B, *ADV* adenovirus, *hMPV* metapneumovirus

RSV was found in isolation or coinfection in 77.1% of patients, rhinovirus/enterovirus in 34.3%, coronavirus in 8.8%, bocavirus in 8.5%, parainfluenza in 7.4%, human metapneumovirus in 5.6%, adenovirus in 4.3% and influenza in 4%. RSV caused 83.4% (146/175) of viral coinfections. The most frequent association was RSV-rhinovirus/enterovirus in 82 children; in 35, a third or fourth virus was found. Table 2 shows the frequency of each of the viruses detected in isolation and coinfection. We observed that RSV was identified more frequently in single infections, while the rest of the respiratory viruses were most commonly found in coinfections. In this regard, coronavirus and bocavirus, which were associated with other viruses in more than 90% of cases, were notable. Figure 2 shows the different viral associations found in the study cohort.

**Table 2:** Absolute number and percentage of positive samples for 17 different viruses or subtypes as single infections or co-infections

Viruses	Single infection n (%)	Co-infection n (%)	Total n = 445
Respiratory syncytial virus	197 (57.4)	146 (42.6)	343
Rhinovirus/enterovirus	49 (32)	104 (68)	153
Coronavirus 229E HKU1 NL63 OC43	2 (5.1) 0 0 1 1	37 (94.9) 3 5 6 23	39
Bocavirus	1 (2.6)	37 (97.4)	38
Parainfluenza Type 1 Type 2 Type 3 Type 4	6 (18.2) 0 0 3 3	27 (81.8) 4 3 11 9	33
Human Metapneumovirus	9 (36)	16 (64)	25
Adenovirus	2 (10.5)	17 (89.5)	19
Infuenza A A/H1 2009 A/H3 B	4 (22.2) 0 1 1 2	14 (77.8) 3 3 5 3	18

The demographic, clinical and treatment characteristics of the two groups of patients with single infections and viral coinfections are shown in Table 3. Viral coinfections were more frequent in girls, while in boys, simple infections predominated (p = 0.003). No statistically significant differences were observed in the remaining demographic characteristics or baseline conditions. When comparing clinical and laboratory data, we found a higher frequency of fever (61.1% vs. 42.6%; p < 0.001) and sepsis (3,4% vs. 0.7%; p = 0.037) and higher levels of C-reactive protein (14.6 mg/L vs. 6.9 mg/L; p < 0.001) in patients with viral coinfections. No significant differences were observed in the results of other complementary tests, antibiotic therapy, or the use of respiratory support.

**Table 3:** Clinical characteristics and treatments of patients with bronchiolitis associated to viral single infection compared with viral coinfection

	Single virus n = 270	Coinfections n = 175	p-value
Sex (%)			**0.003**
Female Male	97 (35.9) 173 (64.1)	88 (50.3) 87 (49.7)	
Gestational age (weeks)	39 [37–40]	39 [37–40]	0.721
Birth weight (grams)	3.1 [2.75–3.47]	3.13 [2.72–3.44]	0.566
Age (months)	2.32 [1.34–4.35]	2.79 [1.45–5.45]	0.074
Weight at admission (Kilograms)	5.16 [4.15–6.23]	5.34 [4.28–6.80]	0.167
Prematurity (%)	42 (15.6)	25 (14.3)	0.765
Type of delivery (% caesarean section)	82 (30.4)	61 (34.9)	0.348
Underlying disease (%)	22 (8.1)	13 (7.4)	0.783
Personal history atopy (%)	19 (7)	14 (8)	0.687
Family history of atopy (%)	48 (17.8)	36 (20.6)	0.489
Siblings (%)	169 (62.6)	120 (68.6)	0.139
Breastfeeding	106 (39.3)	58 (33.1)	0.152
Fever ≥ 38ºC (%)	115 (42.6)	107 (61.1)	**< 0.001**
Food refusal (%)	148 (54.8)	109 (62.3)	0.242
Apnea (%)	16 (5.9)	9 (5.1)	0.726
C reactive Protein (mg/L) n = 424	6.9 [1.4–19.6]	14.6 [4.53–36.1]	**< 0.001**
Urinary tract infection (%)	9 (3.3)	11 (6.3)	0.142
Sepsis (%)	2 (0.7)	6 (3.4)	**0.037**
Antibiotic treatment (%)	90 (33.3)	67 (38.3)	0.286
Consolidation on chest X-Ray n = 260 (%)	33/163 (20.2)	30/97 (30.9)	0.052
Respiratory support (%)			
None Only LFNC oxygen therapy Only HFNC oxygen therapy NIV IMV	28 (10.4) 140 (51.9) 55 (20.4) 44 (16.3) 3 (1.1)	17 (9.7) 85 (48.6) 36 (20.6) 36 (20.6) 1 (0.6)	0.823 0.499 0.959 0.251 1
PICU admission (%)	61 (22.6)	44 (25.1)	0.536
PICU Length of stay (days)	4 [3–6]	5 [3–7]	0.078
Length of hospital stay (days)	6 [4–8]	7 [5–10]	**0.004**

Categorical variables expressed as absolute frequency and percentage, and quantitative variables as median and interquartile range [IQR].

*NPS* nasopharyngeal, *LFNC* Low-flow nasal cannula oxygen therapy, *HFNC* High-flow nasal cannula oxygen therapy, *NIV* Non- invasive ventilation, *IMV* Invasive mechanical ventilation, *PICU* Paediatric Intensive Care Unit

### Viral coinfections and hospital LOS

The hospital LOS was longer among patients with viral coinfections than among those with single infections (median 7 days, IQR 5–10 vs. 6 days, IQR 4–8; p = 0.004). We observed that the duration of hospitalization increased directly in proportion to the number of viruses detected, with a median of 6 days (IQR 4–8) for single infections, 7 days (IQR 4–9) for coinfections with two viruses and 8 days (IQR 5–11) for coinfections with ≥ 3 viruses (p = 0.003).

Cox regression analysis identified factors associated with prolonged hospital LOS (HR < 1). The results of the univariate and multivariate analyses are shown in Table 4. The adjusted model determined that the detection of 3 or more viruses was an independent risk factor for a longer hospital LOS (HR 0.568, 95% CI 0.410–0.785). Other factors associated with a prolonged hospital LOS were the presence of underlying disease (HR 0.578, 95% CI 0.400–0.836), sepsis (HR 0.253, 95% CI 0.117–0.548) and the need for ventilatory support (HR 0.332, 95% CI 0.267–0.413), while older gestational age (HR 1.077, 95% CI 1.033–1.122) and age at admission (HR 1.062; 95% CI 1.035–1.091) were associated with fewer days of hospitalization.

**Table 4:** Risk factors related to longer length of hospital stay. Univariate and multivariate Cox regression analysis

	Univariable analysis			Multivariable analysis		
	HR	95% CI	*P value*	HR	95% CI	*P value*
Sex (male)	0.956	0.870–1.051	0.354			
Gestational age (weeks)	1.073	1.029–1.120	**< 0.001**	1.077	1.033–1.122	**< 0.001**
Birth weight (g)	1.000	1.000–1.000	**0.033**	1.000	1.000–1.000	0.766
Weight at admission (Kg)	1.147	1.093–1.203	**< 0.001**	1.003	0.904–1.112	0.960
Age (months)	1.057	1.032–1.083	**< 0.001**	1.062	1.035–1.091	**< 0.001**
Underlying disease	0.685	0.484–0.971	**0.033**	0.578	0.400–0.836	**0.004**
Prematurity	0.750	0.577–0,974	**0.031**	1.049	0.705–1.562	0.812
Caesarean section	0.951	0.779–1.161	0.620			
Personal history atopy	0.916	0.637–1.319	0.638			
Apnea	0.417	0.275–0.632	**< 0.001**	0.725	0.465–1.130	0.155
Sepsis	0.275	0.129–0.585	**< 0.001**	0.253	0.117–0.548	**< 0.001**
Breastfeeding	0.910	0.748–1.106	0.341			
*Ventilatory support	0.347	0.283–0.425	**< 0.001**	0.332	0.267–0.413	**< 0.001**
PICU admission	0.413	0.329–0.518	**< 0.001**	0.863	0.623–1.196	0.376
RSV	0.883	0.707–1.104	0.275	0.979	0.747–1.283	0.877
Rhinovirus/enterovirus	1.038	0.853–1.262	0.713			
Coronavirus	0.679	0.486–0.949	**0.023**	0.770	0.523–1.134	0.185
Bocavirus	0.802	0.574–1.120	0.195	0.956	0.627–1.457	0.833
Parainfluenza	0.812	0.568–1.161	0.254			
Human Metapneumovirus	0.766	0.511–1.148	0.197	0.740	0.486–1.126	0.159
Adenovirus	1.293	0.815–2.049	0.275			
Infuenza	0.774	0.481–1.243	0.289			
Viral coinfection	0.769	0.635–0.932	**0.007**			
†Viral co-detections						
None			**0.011**			**0.002**
2 Virures ≥ 3 Viruses	0.831 0.644	0.672–1.029 0.474–0.875	0.090 **0.005**	0.858 0.568	0.688–1.071 0.410–0.785	0.176 **< 0.001**

h and 95% CIs are from Cox regression. Bold indicates statistical significance (p < 0.05)

RSV Respiratory syncytial virus

* Ventilatory support includes: HFNC oxygen therapy, NIV and IMV.

† Viral co-detection: Reference group = none

### Viral coinfections and admission to the PICU

Patients with viral coinfections were not more frequently admitted to the PICU than those with a single infection (25.1% vs. 22.6%; p = 0.536). Table 5 shows the results of the univariate and multivariate analyses. The multivariate logistic regression model showed that the independent factors associated with PICU admission were weight at admission (OR 0.821, 95% CI 0.704–0.957), cesarean delivery (OR 1.782, 95% CI 1.060–2.997), presence of apneas (OR 38.16, 95% CI 8.466–172) and RSV infection (OR 3.072, 95% CI 1.470–6.423).

**Table 5:** Risk factors related to UCIP admission. Univariate and multivariable logistic regression analysis

	Univariable analysis			Multivariable analysis		
	OR	95% CI	*P value*	OR	95% CI	*P value*
Sex (male)	1.018	0.653–1.586	0.937			
Gestational age (weeks)	0.893	0.820–0.973	**0.009**	0.978	0.871–1.099	0.405
Birth weight (g)	1	0.999–1	0.348			
Weight at admission (Kg)	0.749	0.649–0.864	**< 0.001**	0.821	0.704–0.957	**0.012**
Age (months)	0.914	0.849–0.985	**0.018**	1.088	0.971–1.219	0.147
Underlying disease	1.541	0.728–3.261	0.259			
Prematurity	1.590	0.898–2.814	0.112	0.683	0.245–1.906	0.464
Caesarean section	1.629	1.032–2.572	**0.036**	1.782	1.060–2.997	**0.029**
Personal history atopy	1.165	0.523–2.594	0.709			
Apnea	47.402	10.96–205.1	**< 0.001**	38.16	8.466–172	**< 0.001**
Sepsis	1.971	0.463–8.388	0.359			
Breastfeeding	1.339	0.849–2.112	0.210			
RSV	2.790	1.460–5.332	**0.002**	3.072	1.470–6.423	**0.003**
Rhinovirus/enterovirus	0.665	0.411–1.076	0.096	0.775	0.381–1.579	0.484
Coronavirus	1.129	0.531–2.401	0.753			
Bocavirus	1.357	0.649–2.838	0.418			
Parainfluenza	0.557	0.210–1.481	0.241			
Human Metapneumovirus	1.024	0.398–2.635	0.961			
Adenovirus	0.858	0.278–2.644	0.790			
Infuenza	1.657	0.606–4.528	0.325			
Viral coinfection	1.151	0.737–1.797	0.536			
†Viral coinfection						
None			0.291			0.188
2 Virures ≥ 3 Viruses	0.979 1.661	0.589–1.626 0.857–3.220	0.934 0.133	0.937 1.843	0.525–1.674 0,879–3.864	0.560 0.068

OR and 95% CI are from Logistic regression analysis. Bold indicates statistical significance (P < 0.05)

*RSV* Respiratory syncytial virus

† Viral coinfection: Reference group = none

## Discussion

The data presented demonstrated that viral coinfections influenced the progression of patients admitted for AB by increasing hospital LOS. The main finding showed a linear relationship between the number of viruses and the length of hospitalization. The detection of 3 or more viruses was an independent factor for a longer hospital LOS. On the other hand, patients with viral coinfections had more frequent fever, higher C-reactive protein levels and developed sepsis in a greater proportion than those with a single infection. However, no significant association was observed between the detection of viral coinfections and the need for admission to the PICU.

Similar to other series [6, 7, 10, 18], in our study, the main etiological agent was RSV followed by rhinovirus/enterovirus, and the most frequent coinfection was RSV-rhinovirus/enterovirus. We observed a high prevalence of respiratory viruses (95%) as well as viral coinfections (39.2%), probably due to the high sensitivity and specificity of the molecular tests used. Consistent with our results, Nascimento et al. observed a viral coinfection rate of 40% among infants with AB seen in an emergency department [7].

In recent decades, molecular diagnostic techniques have led to the discovery of new viruses implicated in AB, such as metapneumovirus in 2001 and bocavirus in 2005 [19–21]. Moreover, their incorporation into clinical practice has increased the detection of viral agents, contributing to improving the diagnostic and therapeutic process of acute respiratory pathology [11, 22]. However, it is speculated that these highly sensitive techniques may detect small fragments of DNA/RNA from viruses that caused previous infections, which would not be responsible for the clinical impact at the time of detection, and there is a risk of overestimating the role of coinfections [12]. In particular, the findings on rhinovirus should be interpreted with caution, as viral RNA has been found in nasopharyngeal samples 4–5 weeks after infection and detected in 12–35% of asymptomatic children. This is less common with other viruses, especially RSV, which has low detection rates in asymptomatic patients (< 5%) [23–25]. Although the presence of respiratory viruses is commonly associated with symptomatology, causality between the detected virus and symptoms is difficult to prove. It would be interesting to analyze viral loads in coinfections to determine the actual active viruses causing the current symptomatology.

The effect of viral coinfections on the evolution of children with lower respiratory tract infection (LRTI) continues to be a controversial topic, with conflicting results in different publications. Similar to our findings, Calvo et al. [8] observed a longer hospital LOS among children younger than 2 years with LRTI and viral coinfections compared to those with RSV infection only (6 days vs. 4.8 days; p = 0.05). In contrast, Petrarca et al. [12], Huguenin et al. [4] and Gil et al. [10] found no differences in length of hospital stay between infants with AB or LRTI with viral coinfections vs. single infections. We do not know the reasons for this disparity in results, although they could be related to certain differences in the molecular techniques used, coinfection rates or sample sizes of the various studies. It should be noted that none of these studies evaluated the relationship between the number of viruses detected and the length of hospitalization, so our results contribute to expanding knowledge in this field with a different approach to the one carried out thus far.

Other factors also associated with the increase in hospital LOS in our study were the presence of underlying diseases, sepsis and the need for ventilatory support, while older gestational age and weight at admission were factors that decreased the duration of hospitalization. It is known that factors related to the characteristics of the patient or the environment are decisive in the genesis of bronchiolitis and establish its progression [1].

The immunopathogenic mechanisms by which different respiratory viruses can cause LRTI with obstructive disorders and wheezing in children are complex. The pathogenesis of lung inflammation induced by any virus, especially RSV, is multifactorial, meaning that in addition to the direct cytopathic effect of the virus on the respiratory epithelium, inflammation mediated by neuronal or nonneuronal mechanisms and innate and adaptive immunity play a primary role [26]. In general terms, respiratory viruses promote the release of inflammatory mediators directly or through substances secreted by cells activated by the viruses themselves, triggering an inflammatory response [27]. We hypothesized that multiple viral infections might elicit a greater inflammatory response than that caused by single infections. Higher C-reactive protein levels and the higher frequency of fever seen in children with viral coinfections from our cohort could reflect this more intense inflammatory response. Although a higher frequency of sepsis was observed in patients with viral coinfections, it was not possible to establish a causal relationship. Franz et al. [28] and Calvo et al. [8] also found more frequent episodes of fever, in addition to increased leukocytosis and antibiotic therapy use among patients with LRTIs and viral coinfections. Despite showing signs suggestive of an increased inflammatory response, the children with viral coinfections in our study were not admitted to the PICU more frequently, nor did they require greater respiratory support. These results coincide with those of the majority of publications in which no association has been found between multiple viral infections and increased admission to the PICU [4, 7, 10, 12].

Concerning other prognostic factors, some authors have described a more severe course of RSV-AB vs. that caused by other pathogens [29, 30]. In our study, we observed that RSV infection was a risk factor for admission to the PICU, although it was not associated with a longer length of hospitalization. On the other hand, we found that infants born by cesarean section were more likely to be admitted to the PICU. Elective cesarean delivery has been described as a predictor of hospitalization for AB [31]; however, to our knowledge, there are no published studies that relate it to a more severe outcome in hospitalized infants, so we highlight this novel finding. Other predictors of severity already described in previous studies [1], such as weight at admission and the presence of apnea, were also risk factors for admission to the PICU.

Among the strengths of this study, we emphasize that the molecular techniques used proved to be highly sensitive in detecting a wide range of respiratory viruses. In addition, the period studied was extensive, which allowed for the collection of a nonnegligible sample of patients during all seasons of the year and in different epidemic seasons, encompassing diverse viral genotypes throughout the years. Due to the substantial variation and reduction in respiratory virus circulation observed during the COVID-19 pandemic [32], we restricted the study period to seasons before the onset of the pandemic. The limitations of this study include its single-center and retrospective nature, as well as the lack of quantitative molecular techniques, which did not allow us to evaluate the activity of the viruses at the time of detection. However, we believe that our results provide useful information for routine clinical practice, since knowing the factors that influence the progression of AB helps to predict its prognosis.

## Conclusion

The results obtained suggest that viral coinfections modify the natural history of AB by prolonging the length of hospital stay in proportion to the number of viruses detected without increasing the risk of being admitted to the PICU. Considering that AB is a complex disease encompassing several pathogenic factors, there is a need for further studies to help expand the knowledge about the direct role of multiple viral infections on the epithelium of the immature lung of the infant, the inflammatory reaction produced and its correlation with clinical severity.

## Data Availability

Data is available on request from first author.

## Abbreviations

AB: Acute bronchiolitis
HFNC: High-flow nasal cannula
HR: Hazard ratio
ICD: International Classification of Diseases
IMV: Invasive mechanical ventilation
IQR: Interquartile range
LOS: Length of stay
LRTI: Lower respiratory tract infection
NIV: Noninvasive ventilation
OR: Odds ratio
PCR: Polymerase chain reaction
PICU: Pediatric intensive care unit
RSV: Respiratory syncytial virus
